# ” Being mutually involved in recovery”. A hermeneutic exploration of nurses’ experiences of patient participation in psychiatric care

**DOI:** 10.1080/17482631.2021.2001893

**Published:** 2021-11-26

**Authors:** Lena Wiklund Gustin

**Affiliations:** a School of Health, Care and Social Welfare, Mälardalen University Sweden, Västerås, Sweden; b Department of Health and Care Sciences, UiT/The Arctic University of Norway, Tromsø, Norway

**Keywords:** Caring science, clinical application research, hermeneutics, mutuality, patient participation, psychiatric care, recovery

## Abstract

**Purpose:**

This study aims at exploring how psychiatric nurses’ experiences of patient participation could be understood from a caring science perspective.

**Methods:**

The design was inspired by clinical application research., which is a hermeneutic approach developed within caring science research. . In this study data were co-created during four reflective group dialogues where five participants’ experiences of patient participation were reflected on in the light of caring science theory and research. The transcribed dialogues were subjected to a thematic, hermeneutic interpretation.

**Results:**

The interpretation gave rise to three themes; giving room for the patient to find his/her own pathway, strengthening personhood, and being in a balanced communion. From these themes an underlying pattern of the meaning of participation as being mutually involved in the patients’ process of recovery arose.

**Conclusion:**

From a caring science perspective the meaning of psychiatric nurses experiences of patient participation could be understood as an interpersonal process reflecting the reciprocity in human relationships. This means a shift in understanding of patient participation from procedures related to the planning of nursing care, to understanding participation as a process focusing on the mutual involvement of patients and nurses in the patients’ process of recovery.

## Introduction

In psychiatric and mental health care an emphasis on person-centeredness and autonomy have contributed to putting patients’ active participation, or patient involvement, on the agenda (Tambuyzer et al., 2011). This is reflected in global (WHO, 2013) and national (Commonwealth of Australia, 2009; HM Government, 2011) health policies around the world, and health care professionals are supposed to enable this participation. Despite legislation and guidelines, a research synthesis reports that patients do not experience that their perspective is sufficiently accounted for (Bee, Price et al., 2015) and that they feel omitted from decisions that not only concern nursing care plans, but their lives (Dahlqvist Jönsson et al., 2015). One possible explanation for this could be that nurses are guided by organizational policies, and that patients and nurses ascribe different meanings to patient participation, leading to different expectations, as well as a failure in obtaining a mutual understanding in relation to patients’ life situations, caring needs and resources (Eldh et al., 2010). As caring science acknowledge patients’ experiences prior to organizational issues it is important to explore how nurses experiences of patient participation could be understood from a caring science perspective. Such understanding could support nurses’ reflections on how they could support patients’ participation.

## Background

Patient participation, or involvement, has been described in terms of the possibilities a patient has to be informed about, or have the possibility to influence, their individual care plans as well as their health and self-care (Eldh, 2006; Dahlqvist Jönsson et al., 2015; Vuokila-Oikkonen et al., 2004). The latter perspective has contributed to an understanding of patient participation that is related to a life-situation, rather than a record about health status and interventions (Eldh, 2019). Participation has also been described in a wider context where patients have been invited as experts in experience, not only to support each other, but also to influence mental health services (Davidson, 2005; Neech et al., 2018; Tapp et al., 2013). This could partly be understood as being related to a change in general nursing ideals from a view of the patient as a passive recipient of care, to an understanding of the nurse-patient relationship as collaborative and ethically motivated (Ashworth et al., 1992; Rush, 2004). Difficulties in establishing a caring relationship built on mutual trust as well as a paternalistic and over-protective attitude from professionals can impede patient participation (Keresi et al., 2019). In contrast, a caring relationship that accounts for patients being simultaneously vulnerable and capable contributes to creating patient experiences of dignity and encourages patients to take responsibility and participate in care. This is in line with research that indicates that patients’ possibilities to be actively involved in their care are limited if the nursing care is task-oriented rather than having a relational focus (Tee et al., 2007; Terry & Coffey, 2019). From this perspective participation is also a matter of being invited to talk about what matters in one’s life, and to feel you are listened to. For example, Koskinen and Lindström (2015) describe how listening can take patients out of their loneliness and enable them to find new and valued directions in life. This appears as pivotal not only for the nurse-patient relationship. It is also important for recovery. Patients who describe that they are able to influence their individual care plans also experience a feeling of being respected, empowered and able to live their lives in a valued way (Goodwin & Happell, 2008b; Sellin et al., 2017a; Tambuyzer et al., 2011). This calls for an invitation to talk about what matters in life and requires openness and humbleness from the caregiver (Koskinen & Lindström, 2015). If this is not at hand and patients fear that the staff should condemn them or conceive of their experiences in a way that brings about negative consequences, there is a risk that the patient’s caring needs are not expressed and thus not accounted for (Blegen, Eriksson, & Bondas, 2016; Wiklund, 2008).

Patients are thus dependent on staff competencies and behaviours, in order to influence their care. However, research focusing on nurses’ experiences describes how nurses experience patient participation in psychiatric care as both important and challenging, as legal restrictions, patient mental health and cognitive impairments have a negative impact on patients’ possibilities, their will and ability to participate (Enarsson et al., 2007; Hörberg, 2008; Magnusson et al., 2020). Other barriers include the nurses’ own lack of time and inadequate psycho pedagogical education (Goodwin & Happell, 2008a). Nurses can also experience insecurity and be afraid of losing control, and conflicting values among staff has also been described as having a negative influence on staffs’ willingness to involve patient in psychiatric care (Enarsson et al., 2008; Glenister, 1994). Moreover, health care professionals value their professional accountability in a way that might conflict with the philosophical underpinnings of patient participation (Bee, Brooks et al.).

More than 25 years ago Glenister (1994) described that participation and social interactions should not be separated. However, there are still different discourses in mental health nursing and psychiatric services, where concepts like participation and user involvement is used differently within a government discourse, among mental health nurses, and also by service users (Hui & Stickley, 2007; Zeeman & Simons, 2011). This contributes to different understandings and approaches to patients participation. As concluded by Hui and Stickley (a.a.) nurses need to be aware of these differences, and the different distribution of power that is associated with them. Such reflections are necessary, not only to ensure patients’ active involvement in care, but also to transform psychiatric nursing practice towards more democratic, person-centred and recovery-oriented mental health practice (Borg et al., 2009; Storm & Edwards, 2013). As discourse, and thus theoretical standpoints, appear as vital for how meanings are ascribed to patient participation, and thus on how they are realized in clinical work, this study aims at exploring how psychiatric nurses’ experiences of patient participation could be understood from a caring science perspective.

## Methodological approach

The methodological approach was inspired by Lindholm et al.’s (2006) as well as Koskinen and Nyström (2017) writings on clinical application research. They describe clinical application research as a participatory research grounded in a hermeneutic, interpretive tradition. A key issue is that knowledge and understanding is developed in a reciprocal process where data-generation is linked to reflective group dialogues (RGDs) where practice is linked to theory. This contributes not only with research data, but also supports co-researchers’ practice. This was considered an advantage as the unit where the study was undertaken was in a process of organizational change. The objectives in the organization were to increase patient participation, and to develop a caring culture reflecting basic assumptions in the Scandinavian tradition of caring science (Arman et al., 2015). Thus, the methodological approach did not only contribute with qualitative data to the present study, but was also considered as an opportunity to improve clinical work.

### Participants as co-researchers

This approach is interesting as the researcher, called the principal investigator, and the participants—or the co-researchers, which is the concept used within this tradition—co-construct data during joint reflections. The naming of participants as co-researchers presents them as valuable scientific resources who have an active role in developing new understandings (Lindholm et al., 2006). All professionals working on the unit were informed about the project, and the nurses were invited to a series of four RGDs. Five of the eight nurses working on the unit, one man and four women, volunteered. Their nursing experience varied between two years and more than twenty-five years.

### Pre-understandings and theoretical perspective

The study was undertaken at a small psychiatric unit in a rural area. As a result of a survey that revealed that the patient did not experience that they had enough influence on the care given, patient participation was a prioritized area. However, there was also an assumption that the official standpoint that mainly focused on information and shared decision making was narrow and not in line with the unit’s value base and intention to ground their nursing care in caring science. They had an articulated value base rooted in Eriksson’s theory of Caritative Caring (Lindström et al., 2017), and the co-researchers expressed a specific interest in broadening as well as deepening their theoretical understanding of how patient participation could be linked to caring science. The principal investigator has a background as a psychiatric nurse. As a researcher she is rooted in caring science and has collaborated with caring science researchers from Scandinavia as well as other parts of the world.

### Data generation

The reflective group dialogues can be described as a continuous hermeneutic process between theory and practice, as new understandings occur in a cyclic process of interpretation between the principal investigator and co-researchers. A part of the principal investigator’s role is to create a hermeneutical room, a joint space where different horizons of understandings be expressed (Koskinen & Nyström, 2017). This requires an open and trustful atmosphere that allows people to admit not only their positive experiences but also their shortcomings (Nyholm et al., 2018). This was accomplished by making it explicit that we were struggling together to explore participation from our different horizons, and that the co-researchers’ experiences as practitioners were just as important as the researcher’s theoretical knowledge.

The co-researchers and the principal investigator (i.e. the author of this article) met for an RGD once a month for four months. Each dialogue was digitally recorded and lasted about three hours (including a break of twenty minutes). They were organized around different themes (Table I) that unfolded between the RGD as the co-researchers were attentive to issues relating to patient participation in their clinical work. This was not related to standardized observational procedures, rather it was a matter of paying attention to situations in their daily work where patient participation was on the agenda. When possible, the co-researchers reflected together with other professionals who were present in the situation, about what have just happened and what kind of thoughts and feelings that have guided their actions. These experiences produced rich and vivid data, which were explored and reflected on in the light of theory. These were introduced into the reflective dialogues by the principal investigator. Thus, each session added new research data while simultaneously supporting the co-researchers’ integration of theory and practice.

**Table I.** Overview of reflective group dialogues

Reflective Group Dialogue	Focus	Theoretical input
#1	Reflections on good nursing care in relation to the unit’s documented value base, focusing on alleviating suffering and promoting dignity, and how that could be related to patients’ participation.	Eriksson’s theory of caritative caring (Lindström et al., 2017) (Eriksson, 2006). Health seen as experiences of being able to fulfil one’s minor and major projects in life (Todres et al., 2014) Psychiatric ill health seen as “problems in living” (Barker & Buchanan-Barker, 200).
#2	What is the meaning of participation from the perspectives of patients and relatives	An interview study presented by a student prior to the present project, describing how patients on the unit experienced their care. Research on patients’ and their close ones’ experiences (Eliacin et al., 2015, (Sellin et al., 2017a, Sellin et al., 2017b)
#3	Participation as an existential and relational phenomenon	Caring encounters seen as sharing a space of togetherness (Holopainen et al., 2014), and the caring relationship seen as encompassing a mutual connection, becoming touched by the other, and creating a story together as well as the nurse’s obligation to protect patient dignity (Kasén, 2002).
#4	Application of new understandings	How to establish experiences of participation such as being involved in community with others and finding possibilities to advocate and support patients in relation to other professionals.

In contrast to a traditional focus group interview, the researcher has a more active role in introducing theory and supporting the co-researchers’ reflections on their experience. This is in line with an understanding of philosophical hermeneutical interviews being joint reflections on the meaning of experiences that enable a co-created understanding, (Geanellos, 1999; Vandermause & Fleming, 2011) and with Guzys et al. (2015) and their adaption of philosophical hermeneutics in Delphi-studies.

### Ethical considerations

The co-researchers were informed about the aim of the study, about the procedures, that participation was voluntary, and that they could withdraw from the study at any time. In keeping with the clinical application research intention of contributing to care development, co-researchers agreed to safeguard confidentiality by not disclosing who had said what to other people. However, they were encouraged to discuss patient participation with all colleagues. This was also ethically motivated, as it promoted reflections about the care given and is thus in line with the explicit ethos of clinical application research (Koskinen & Nyström, 2017). The researcher had an ethical responsibility to guide the reflections so that different voices and perspectives were acknowledged, and to report the project without violating confidentiality. The project was approved by the local ethical board.

### Data analysis

All RGDs were transcribed verbatim and subject to a hermeneutic interpretation, influenced by the philosophical writings of Gadamer (1989), as well as the Gadamerian-based method described by Fleming et al. (2003). According to these philosophers’ writings, interpretation is an act of understanding what arises in the encounter between the reader and the text. This implies a dialectic movement between the text as a whole, in other words the transcript from all the reflective group dialogues, and its parts in the form of each transcript, as well as smaller units within these texts. Hence, after repeated readings of the transcriptions to gain an overall understanding, the text was subject to a thematic structural analysis where the transcribed texts were divided into meaning units and condensed to articulate their essential meaning. Next, the condensed meaning units were reflected on regarding differences and similarities. In line with Fleming et al. (2003) these were articulated as themes and subthemes. These were related to each other to describe an underlying pattern of meaning which was formulated as a main theme. As theory was constantly present during the RGDs, and the aim of the study was to explore how psychiatric nurses’ experiences of patient participation could be understood from a caring science perspective, caring science theory and research is used to elaborate the interpretations in the presentation of findings. Thus, caring science theory has been integrated throughout the research process,

The aim of the study was to explore how psychiatric nurses’ experiences of patient participation could be understood from a caring science perspective. Hence, as caring science theory was constantly present during the RGD, it is therefore also integrated in the presentation of findings.

## Findings

In the interpretations of patient participation in psychiatric care, the fact that human beings are mutually related to each other, contributes to how meaning is ascribed to the co-researchers’ experiences. Hence, during the RGD the nurses agreed with Holopainen et al.’s (2014) description of caring encounters as well as with Kasén’s (2002) view of caring relationships which encompasses a mutual connection, becomes touched by the other, creates a story together and places responsibility on the nurse to protect patient dignity.

From the nurses’ perspective, patient participation cannot be reduced to just offering patients an opportunity to participate in decisions about their care. Nurses also need to become more involved as persons in the caring encounter with the patient and, as Kasén (2002) describes it, become touched by the patient. It is from this encounter that a joint understanding about the patient’s suffering, their caring needs as well as their visions in life can arise. Involving patients in care is, to use Kaséns words, a matter of creating a story together, not only a care plan. In other words, it is not only patients who participate in care, nurses need to be engaged and present as well. Holopainen et al. (2014) describe this as an aspect of mutuality and as a way of being together in a way that gives life’s mystery a possibility to shine forth and become understandable for the encountering persons. From an understanding of participation as mutual relatedness, the patient must be understood as a unique Other, who the nurse strives to understand. However, as described by Todres et al. (2014), even though nurses strive to “reach towards insiderness” they simultaneously need to remain in a “not knowing” position. This is includes tolerating the frustration associated with not being able to fully understand, and avoid taking control over the other to relieve own feelings of shortcomings. In relation to patient participation this is important, as “taking control over” might have a negative impact on the patient’s experience of dignity. Furthermore, even though having another person (for example, a nurse) controlling one’s life might be experienced as a relief for a short periods it can undermine experiences of health as being capable of fulfiling minor and major projects in life and living a meaningful and valuable life. In contrast, if nurses are able to co-create a space of togetherness with the patient, the person is supported to reclaim the direction in his/her own life, which according to Barker and Buchanan-Barker (2005, 2010) is the essence of mental health recovery.

### Main theme: Being mutually involved in recovery—participation as a double helix

Hence, the main theme describing participation in psychiatric care, could be formulated as “being mutually involved in recovery”. Participation as mutual involvement can be described as a double helix, having both an outer form expressing the “how” (the sub-themes), and an inner form, expressing the “what” (the themes) of participation. These will be explained in more detail below. While the outer form became visible through different intentions and related actions undertaken to account for patients’ participation, the inner form can be described as a participatory culture. Rather than focus patients’ participation in decisions related to nursing care plans, a participatory culture is characterized by nurses and patients being mutually engaged in patients’ recovery processes. This was shown when one of the co-researchers expressed her understanding during the last reflective group dialogue.

You know, we can invite the patient to a joint meeting and make up a care-plan together, but it is usually very much on our terms. We tell the patient about different opportunities, and the patient is encouraged to choose among them. But real participation is something else, there is an experience of being in it together which makes me more engaged too (Nurse 5).

An overview of themes and subthemes is presented in Table II. In the following presentation of themes (headings) and subthemes (*italics*) the interpretation above of participation as being mutually involved in recovery is the whole, towards which the parts are reflected before additional links to theory are made.

**Table II.** Overview of themes and subthemes generated in the thematic structural analysis

Themes	Subthemes
Giving room for the patient to find	Sharing time together
his/her own pathway	Showing a genuine interest
	Negotiation of mutual goals
	Being flexible
	Carrying both one’s own and patients’ disappointments
Strengthening personhood	Accounting for the patient’s personal strengths and values
	Involving important others
Being in a balanced communion	Accepting the other person’s invitation
	Building mutual trust
	Taking responsibility

### Giving room for the patient to find his/her own pathway

This theme implies a shift from experiences of participation seen as motivating patients to comply with nurses’ and other care-givers’ perspectives, towards an understanding of participation as supporting patients to strive towards their own values. In this theme mutuality is related to nurses and patients *sharing time together* and *showing a genuine interest* in each other. In this space of togetherness, it is assumed that the patient experiences him or herself as being involved in a caring community, not only because of receiving care and confirmation of suffering, but by being able to contribute to a shared narrative.

I think, that when I’m able to show that I appreciate the patient’s company, for example, if we are preparing the evening tea and coffee in the kitchen, that too is a way to make him (the patient) more involved. We can use this time not only to prepare the sandwiches, but also to … to build something. You know, we have this small talk and share the moment and maybe we discuss his issues, but he could also feel that I need his assistance, that he is part of what is going on (Nurse 4).

Hence, being together in an ordinary situation, where patient and nurse both contribute on equal terms, and where the patient feels appreciated, can support the patient in rediscovering and strengthening personal resources that can be adapted to daily life. Simultaneously, these moments can provide a “scene” where it might feel more relaxed to talk about what matters to the patient. Such occasions also contribute to experiences of being mutually involved during patient recovery processes,, and might be memorable moments to refer back to. For example,

patients might say things afterwards like ‘do you remember when we made the apple-pie’, and I can answer ‘Yes, I do. And I think everybody else does too. You really put a silver lining on that rainy day”’ (Nurse 4).

What became evident was the reciprocity of these aspects of participation—patients also invested time and interest in nurses in a way that was not only appreciated by the nurses, but was also used to encourage and validate the patient. Reciprocity and mutual understanding are also important when more challenging issues are to be discussed, for example, when reflecting on the *negotiation of mutual goals*.

I can’t support a goal that I know would bring about chaos in the patient’s life. So, we must find out what it is all about. I need to understand the patient, but he also needs to understand why I’m not supporting everything. Then we can break it down to something we can do together (Nurse 3).

Envisioning patient participation as allowing the other to find his/her own pathway also means *being flexible*, as “it is impossible to really know what is best for them. We think we know, but if we try to make them fit into our set patterns of how it ought to be, they will fall out of them as soon as they leave here” (Nurse 1). When mutuality is associated with being touched it also implies a preparedness to *carry both one’s own and patients’ disappointments* when encountering setbacks or failures.

You know, you can’t say things like “what did I say, I knew this would happen” when a person fails, even if that is what you feel. You can show that you share their disappointment and grief, but you must never rub it in, never. That makes it worse, even if you think that this was not surprising. And then, I guess, it is even harder to go on together [—] But you can’t pretend as if nothing has happened either. You must be there for them … and give comfort and hope about success the next time (Nurse 1).

Nurses thus need to be emotionally available, and as described by Kasén (2002) allow themselves to become touched by the patient, and to disclose this for the patient as a sign of mutual connectedness. In line with Todres et al. (2014) the genuine interest in the other person could be understood as “reaching towards” the other, rather than as striving towards “knowing about” (a.a. p. 7) the other or having complete understanding. This also implies that participation is associated with an intention to establish common goals based on connectedness and respect for the other person’s otherness and infinity, rather than on compliance.

### Strengthening personhood

The reciprocity in relationships also contributes to the strengthening of personhood. This not only indicates a shift in focus from the patient to the person, but in contrast to a traditional medical paradigm with focus on compliance and symptom reduction, this understanding of participation as meaning involvement, highlights psychiatric nursing care as being directed towards supporting recovery by *accounting for the patient’s personal strengths and values*.

I really think that we must see what works too, and not only the suffering. It is of course important to alleviate it, but I don’t think that is enough. And sometimes it is rather depressing too, to focus on all the problems and everything that is chaotic and so on in their lives. Because if we only focus on that, then that is what they present for us … I think it can be as if they are afraid of talking about their joys rather than, that they think that we are not interested in that (…) or even worse, that we will discharge them before they are prepared for it if we think that they are ‘too happy’. We must make them believe that it is OK to be happy, to succeed with things and still get our support when they need it (Nurse 3).

As described by Barker and Buchanan-Barker (2004), being seen as a person and being able to express one’s story, are pivotal in recovery. This implies that the patient’s opinion on what counts in life is important, and this includes the *involvement of important others*. Significant other people are part of a person’s story and involving them not only as sources of information but as links to ordinary lives, appears as important in relation to personhood. In line with Sellin et al. (2017b) the nurses also reflected on this as being valuable for the important others, as they too could be validated and acknowledged in a new way.

When they’re here, they are kind of lifted away from reality. It’s like a sheltered workshop here, and really … that does not make them fit for life. That’s why we should involve families and friends, they can teach us … and they can remind the patient about what matters (Nurse 2).

Involving significant other people is understood as a means of reconnecting to the world outside the psychiatric settings, and thus make use of patient strengths and resources.

This is in line with the theoretical perspective’s view of respecting human dignity, although it also challenges existing roles and routines where hospitalized patients by tradition are supposed to spend most time on the ward together with professionals. As stated by one of the nurses towards the end of the project,

I have spent so many years striving to resolve their problems, alleviating their worries, and making things as easy as possible for them. And now I should take a step back, sit on my hands. Trust that they, deep inside, know what works for them. That is hard. As with your children, you want to protect them, and you think that you know what’s best for them. But you don’t – at least not all the time” (Nurse 1).

Such reflections about new roles and ways of caring also contribute nuances to the view of nursing care as focusing on alleviating suffering (Eriksson, 2006). The shift towards recovery also meant that patients’ resources and personal strengths were given more room, and nurses’ problem solving and planning less. This does not mean that nurses should not alleviate suffering, rather that they need to be more attentive to patients’ own strategies for enduring suffering so that they do not inflict further suffering in their efforts to “do good” (Madjar, 1998; Morse, 2001; Vincze et al., 2015).

### Being in a balanced communion

Participation as being mutually involved in recovery is also based on nurses and patients *accepting the other person’s invitation* to a common space where one relates to each other as persons, not through the roles of nurses and patients. This is reflected in this dialogue between participants.

Nurse 5: I think that I have been … in a way … not so responsive for them from time to time. I have introduced myself and told them that I am their contact nurse and said, “you know where to find me” and things like that. But it has been more of a routine. Of course, if they appear in the doorway (to the nurses’ office) or ring the bell, I answer, but if they don’t I think that they have better trust in somebody else.

Nurse 4: But that is how it is, you can’t force them to talk with you.

Nurse 5: Yes, I know, but I mean that I haven’t been very welcoming so to say, and I have left the responsibility to them. Especially if I have been thinking about (mentions problems with a relative regarding private life). And then, if they make indirect contact, for example, by asking for something I have just provided them with that extra medication or pillow or whatever.

Nurse 4: You have done your best, I think. Just as the patients, but sometimes people make mistakes. But you are right, it is not always easy to notice that they want something more. But other times it is obvious instead, even if it is in a way that might be scary.

Nurse 3: And that is really challenging to see that they want something, because it could look like the opposite.

Being responsive can be challenging, but if the nurse can balance the power relations and the inevitable asymmetry as described by Fredriksson and Eriksson (2003), it can contribute to the *building of mutual trust*. This means that to support participation, nurses also “need to trust the patients, and believe that they mean what they say, even if we think that it is unrealistic (that they should manage to stop taking drugs)”. Such mutual trust is considered essential for what Watson (2008) describe as helping-trusting relationships.

Even though nurses need to acknowledge their own vulnerability and admit their own shortcomings, they are also responsible for communicating it in a way that does not put the burden or blame on other people.

Sometimes you need to go against the rest (of the staff), just to be able to look the patient in the eyes. And yourself as well. If you don’t, then you are as they say in Bröderna Lejonhjärta (Book by the author Astrid Lindgren) only a little shit (Nurse 1).

This is interpreted as *taking responsibility*, which is grounded in caring ethics and states that the nurse is reliable and competent, but also has the courage to take sides with patients and make their voices heard. Hence, than a formal, professional responsibility this is a matter of an ethical demand that appeals for a personal responsibility linked to the ethos of caring science (Gabrielsson et al., 2016; Hemberg & Kaarre, 2016; Wallinvirta, 2011).

## Discussion

Participation when viewed as mutual involvement can be described as a double helix, having both an outer form expressing the “how” (the sub-themes), and an inner form, expressing the “what” (the themes) of participation. Understanding patients’ participation as having an outer and an inner form can shed light on some of the difficulties with patient participation in psychiatric care. It can be assumed that the pre-understanding the participants had about the organizational view on participation as focusing information and decision-making is transferable to other settings as a too task-oriented approach is put has been put forth in the literature as obstructing patient participation (Bee et al., 2015; Dahlqvist Jönsson et al., 2015; Terry & Coffey, 2019). Thus, an organizational perspective could contribute to a pre-dominant focus on the outer form and a strive to “produce” participation by performing different task. However relevant these tasks may be, they might be insufficient if patient participation is understood from a caring science perspective. From this perspective the double helix could be understood in the light of Lindström’s (1994) description of caring intentions as the “what” of psychiatric caring, and “caring activities” as the how. This means that they cannot be separated from each other, as every how needs to be motivated by a specific intention that supports the patient’s health-processes and recovery. Therefore, patient participation cannot be reduced to specific interventions such as being transparent with documentation, allowing time for joint planning, and giving the patient possibilities to make informed choices. These kinds of interventions can be valuable “tools”, but are not sufficient by themselves to create a participatory culture.

The findings highlight the fact that giving room for the patient to find his/her own pathway is one of the inner aspects of participation, and is closely related to caring encounters as described by Kasén (2002) and Holopainen et al. (2014). The nurses’ intentions of getting to know the other and negotiating mutual goals is close to patients’ descriptions of shared decision-making being built on relationships rather than procedures (Eliacin et al., 2015). However this goes beyond decisions, as a profound sense of community as well as a sense of being valuable is closely related to the sense of togetherness that can emanate when time is spent together for other reasons than care-planning (Molin, 2019). Furthermore, the negotiation of goals is not just a matter of motivating the patient to comply to goals suggested by the caregivers. Rather, it is related to support recovery and strengthening personhood by accounting for personal resources. This is in line with an understanding of person-centred care as focusing meaningfulness rather than functionality (Håkansson Eklund et al., 2019). This also implies that patients themselves are the experts in relation to what matters and what might be applicable in their own lives, while professionals are supposed to be knowledgeable about possible solutions on a general level. As described by Larsson and Jormfeldt (2017, p. 2) “sharing knowledge and expertise between persons is a key component of mutuality” and closely related to interdependence. This is in line with researchers like Delmar (2012), Eckerström et al. (2019), and Finfgeld (2004), who imply that nurses need to give up some of their control to reduce differences in power and that nurses’ power should be used to support patient recovery, rather than ensuring compliance and certain behaviours. The latter has been described not only in forensic psychiatry which by its nature is strictly regulated (Hörberg, 2008) but also in general care (Rundqvist, 2004).

The caring communion has theoretically been described as a “space of togetherness” (Holopainen et al., 2014), and in this study this space provides the prerequisites for participation as grounded in mutuality. This has clear links to Fredriksson and Eriksson (2003) and their claim that nurses have the responsibility to balance the inevitable asymmetry in caring relationships. They also write that there is a risk that nurses who lack self-esteem and autonomy might comply with others, patients or peers, to stay in control and feel appreciated for their kindness. However, mutuality is not a matter of always being kind. Rather, it is a matter of being aware that one’s own acts as a nurse affect patients’ understanding of themselves and their opportunities, and to be together with others in a responsible way based on this premise. This is like Donner and Wiklund Gustin (2020) conclusion that nurses need to have the courage to remain in uncertainty and openness to apprehend the patient’s caring needs. s this study demonstrates patient participation is not only about a shift in power, but also a shift in the focus of nursing care—from nurses’ interventions to patients’ lives.

The shift in understanding of patient participation from procedures related to the planning of nursing care, to understanding participation as being mutually involved in the patients’ process of recovery was a new insight for the co-researchers and their colleagues. This calls for reflections on whether the good intentions underpinning regulations and recommendation about increased patient participation might be overrun if health-care professionals strive to apply participation as a nursing intervention without reflecting on interpersonal aspects of participation. This is in line with important notions made by Angel and Frederiksen (2015) in their review of how patient participation is addressed in empirical studies in a variety of clinical contexts, including psychiatric care. They concluded that participation in its ideal form is impossible to achieve because of the unequal relationship between patients and professionals. Patients participation possibilities need to be understood as embedded in social structures, and cannot achieve optimal levels unless there is enough time to build relationships and share knowledge. This study adds nuances to Angel and Frederiksen (2015) findings, by claiming that the relationship between the patient and the nurse is not just a prerequisite for patient participation. On an existential level, relatedness and mutuality could be understood as being a part of patient involvement in care.

### Methodological considerations

In line with Fleming et al.’s (2003) Gadamerian approach trustworthiness is related to the process rather than to the conclusions. This implies that the pre-understandings of both the co-researching participants and the principal investigator must be challenged during the process. In this study the theoretical perspective was made explicit and used during the RGDs. This was in line with the hospital’s ambition to frame nursing interventions within a caring science paradigm but could be questioned as a probable source of bias, as it implies that the principal investigator could have an impact on the content of the RGD. The hermeneutic circle as a movement between the whole and the parts, and from preunderstanding to renewed understanding becomes visible in the thematic analysis. Focusing on the text as parts is an act of decontextualization from the context of the RGD, as well as from the theoretical perspective, before uniting themes and theory in the interpreted presentation of findings (Debesay et al., 2008). In a full-scale clinical application project, it is common that the co-researchers are also involved in interpreting the transcribed text. Due to limited time for the co-researchers this was not the case in the present study, and the principal investigator performed the analysis.

In addition, in clinical application research, implementation and development of theoretical knowledge is in focus (Lindholm et al., 2006). Theory should not be directly absorbed by the clinicians, but be subject to joint reflections that allow for a suspense between what is already known and the unknown. This allows for new understandings that can facilitate new actions (Koskinen & Nyström, 2017). Hence, in this project the findings from the analysis reported in this article, were jointly reflected on not only with the co-researchers, but also with all health care professionals as well as the managers at the clinic. These reflections led to considerations about how the care could be developed to account for the renewed understanding of participation. This process will be further described and reported on separately.

It could be argued that the wish to develop knowledge that is immediately applicable in the context of its origin is a limitation in regards to transferability, as well as the relatively few participants. However, in line with Lindholm et al. (2006)it is argued that the theoretical understanding that arose is transferable to other contexts. Even though this project was undertaken in a small unit, patient participation in nursing care is generally an area of importance. Furthermore the number of co-researchers is not the key issue, rather it is the way that their experiences is represented in the findings that contribute to the value of a hermeneutic study. This is in line with a hermeneutic understanding of transferability as an opening of new, possible horizons of interpretation (Fredriksson, 2014).

## Conclusions

From a caring science perspective the meaning of psychiatric nurses experiences of patient participation could be understood as an interpersonal process reflecting the reciprocity in human relationships. This means a shift in understanding of patient participation from procedures related to the planning of nursing care, to understanding participation as a process focusing on the mutual involvement of patients and nurses in the patients’ process of recovery. Most significantly, to develop psychiatric care the meaning of patient participation needs to be further reflected on with respect not only to shared decision-making and planning, but to experiences of nurses as being in a space of togetherness where they and the patients are mutually engaged to support the patient’s process of recovery.
